# MOTS-c partially protects against skeletal muscle deterioration in C26 cachexia

**DOI:** 10.3389/fmed.2026.1838178

**Published:** 2026-05-25

**Authors:** Nicholas A. Jamnick, Patrick D. Livingston, Caleb J. Gammon, Natalia M. Weinzierl, Leah J. Novinger, Andrea Bonetto

**Affiliations:** 1 Department of Pathology, University of Colorado Anschutz, Aurora, CO, United States; 2 Cancer Center, University of Colorado Anschutz, Aurora, CO, United States; 3 Colorado Nutrition Obesity Research Center, University of Colorado Anschutz, Aurora, CO, United States

**Keywords:** cachexia, colorectal cancer, MOTS-c, muscle, protein homeostasis

## Abstract

**Background:**

Cancer cachexia is a multifactorial metabolic syndrome marked by progressive skeletal muscle loss, reduced function, and increased mortality. Mitochondrial dysfunction is a key driver of this phenotype. MOTS-c, a mitochondrial-derived peptide that regulates metabolic homeostasis and mimics exercise signaling, may counteract cachexia, but its role remains largely unexplored, and human studies using MOTS-c in subjects with cancer cachexia are needed.

**Methods:**

Differentiated myotubes were treated with MOTS-c (50 μM) to assess intracellular signaling. *In vivo*, male mice were inoculated with Colon-26 (C26) carcinoma cells and treated daily with MOTS-c (15 mg/kg/2x Day, i.p.) or vehicle. Body weight was monitored daily. At euthanasia, organ and skeletal muscle masses were measured. Molecular analyses focused on FOXO signaling, atrogene expression (MuRF1, Atrogin-1), and mitochondrial biogenesis markers, including PGC-1α.

**Results:**

*In vitro*, MOTS-c increased PGC-1α mRNA (+84.6%) and AMPK phosphorylation (+103.1%). C26 tumor-bearing mice exhibited significant systemic wasting (~9% body weight loss). Although MOTS-c did not prevent total body weight or fat loss, it significantly preserved skeletal muscle mass, rescuing quadriceps weight (+12% vs. C26 vehicle; *p* < 0.05) and trending toward protection of gastrocnemius mass and EDL function. Cachexia-induced upregulation of Atrogin-1 (+8.6-fold) and MuRF1 (+16-fold) was attenuated by MOTS-c, accompanied by increased inhibitory pFOXO1 (+80%), reduced pFOXO3a (−39%), and partial restoration of PGC-1α protein (+143%).

**Conclusion:**

Our findings demonstrate that MOTS-c partially protects against skeletal muscle loss in C26 cachexia by modulating FOXO-driven catabolic signaling and promoting mitochondrial biogenesis, supporting its therapeutic potential in cancer cachexia.

## 1 Introduction

Cachexia is a multifactorial syndrome characterized by unintentional weight loss, reduced skeletal muscle mass and function, impaired tolerance to chemotherapeutics, decreased quality of life, loss of free fat mass, and widespread metabolic dysfunction (1–3). Up to 80% of adults with advanced cancer will develop cachexia, and approximately 30% of affected individuals will die directly from this condition (1). Tumor-derived pro-inflammatory cytokines drive muscle wasting by suppressing protein synthesis, enhancing protein degradation, and disrupting mitochondrial homeostasis, ultimately producing the severe clinical manifestations of cachexia (1, 4).

Currently, there are no FDA-approved therapies to treat cachexia in the United States. Several drugs, including mitochondria-targeted antioxidants (e.g., MitoQ, SkQ1, SS-31) have been tested to attenuate cancer induced cachexia in pre-clinical mouse models with promising results (5–9). These compounds act by mitigating mitochondrial dysfunction: MitoQ accumulates in the inner mitochondrial membrane where it is reduced to an active antioxidant that protects against lipid peroxidation (10); SkQ1 similarly prevents the peroxidation of cardiolipin and inhibits the formation of superoxide (11); and SS-31 is thought to attenuate mitochondrial ROS production (12). Altogether, these agents partially alleviate cachexia-related impairments in mice inoculated with Colon-26 (C26) carcinoma cells, a widely used and well-characterized model for the study of cancer cachexia (5–9).

A recently identified mitochondrial microprotein, mitochondrial open reading frame of the 12S ribosomal RNA type C (MOTS-c), has emerged as potential therapeutic for multiple metabolic and musculoskeletal diseases. MOTS-c is a 16-amino acid microprotein encoded in a small open reading frame within the 12S rRNA region of the mitochondrial DNA (13). Since its identification in 2015, MOTS-c has been shown to regulate metabolism, where exogenous administration prevents metabolic disorders (13–15), attenuates skeletal muscle atrophy (16, 17), promotes mitochondrial biogenesis in skeletal muscle (13, 18), improves bone health (19–21), repairs myocardial damage (22), and maintains exercise capacity and median lifespan (18). Under metabolic stress (e.g., exercise), endogenous MOTS-c translocates from the mitochondria to the nucleus to modulate adaptive nuclear gene expression (23), whereas exogenous MOTS-c directly bind to and activates protein kinase CK2 (24). These pre-clinical findings highlight the therapeutic potential of MOTS-c in treating disorders involving skeletal muscle and mitochondrial dysfunction.

To date, no studies have examined whether MOTS-c can preserve skeletal muscle in the context of cancer cachexia. Understanding whether MOTS-c can be used to mitigate the cachectic phenotype is therefore of significant importance. Here, we aimed to investigate whether exogenous MOTS-c attenuates cancer-induced cachexia in mice bearing C26 tumors.

## 2 Materials and methods

### 2.1 Animals

All experiments were conducted with the approval of the Institutional Animal Care and Use Committee at the University of Colorado Anschutz, Aurora, CO and were in compliance with the National Institutes of Health Guidelines for Use and care of Laboratory Animals. In order to investigate the effect of MOTS-c on musculoskeletal health in cachectic animals, 12-week-old CD2F1 (purchased from Inotiv, West Lafayette, IN, United States) were randomized into four group: controls (*n* = 5), MOTS-c (*n* = 5), C26 (*n* = 8) and C26 + MOTS-c (*n* = 8). Sample size determination required a minimum of *n* = 6 per experimental group to achieve adequate statistical power (*β* = 0.80, *α* = 0.05), based on effect-size calculations (*d* = 1.20) derived from previously published studies examining skeletal muscle mass (17). Tumor hosts were subcutaneously inoculated with 1.0 × 10^6^ C26 colon adenocarcinoma cells, similar to (6, 25). MOTS-c (15 mg/kg) (GenScript Inc., Piscataway, United States) was administered intraperitoneally (IP) the same day as tumor inoculation and twice daily until sacrifice (~day 14). Dosage was based off previously published research (17). Control (*n* = 5) and C26 (*n* = 8) mice received an equal volume of vehicle (sterile saline) injected subcutaneously. Mice were monitored and weighed daily. At endpoint (after 14 days), the animals were euthanized under light isoflurane anesthesia. Skeletal muscles (gastrocnemius, quadriceps, tibialis anterior) and organs (heart, liver, spleen, kidneys and epididymal fat) were harvested, weighed, snap frozen in liquid nitrogen and stored at −80 °C for further studies. The tibialis anterior muscles were frozen in liquid nitrogen-cooled isopentane for histological assessments.

### 2.2 Western blotting

Total protein extracts were obtained homogenizing 100 mg quadriceps muscle tissue in radioimmunoprecipitation assay (RIPA) buffer [154 mM NaCl, 1.0% NP-40, 0.25% sodium deoxycholate, 0.1% sodium dodecyl sulfate (SDS), 1 mM ethylenediaminetetraacetic acid, and 65.2 mM Tris, pH 7.4] completed with 5 mM Na_3_VO_4_, 0.4 mM NaF, 0.2 mM MG-132 Proteosome Inhibitor (NC2684146, Invivogen, Carlsbad, California, United States) 0.02 mM phenylmethylsulfonyl fluoride, and protease inhibitor cocktail tablet (Roche, Indianapolis, IN, United States) and (Thermo Scientific, Waltham, MA, United States). Cell debris were removed by centrifugation (15 min, 18,000 g), and the supernatant was collected and stored at −80 °C. Protein concentration was determined using the bicinchoninic acid (BCA) protein assay method (Thermo Scientific, Waltham, MA, United States). Protein extracts (15–40 μg) were then electrophoresed in 4–15% gradient SDS Criterion Tris–HCl precast gels (Bio-Rad, Hercules, CA, United States). Gels were transferred to nitrocellulose membranes (Bio-Rad, Hercules, CA, United States). Membranes were blocked with 5% Bovine Serum Albumin (BSA) in TBS-Tween (0.1%) at room temperature for 1 h, followed by an overnight incubation with diluted antibody in Tris Buffered Saline-Tween (TBS-T) (0.1%) at 4 °C with gentle shaking. After washing with TBST-T the membrane was incubated at room temperature for 1 h with either Anti-rabbit IgG (H + L) DyLight 800 or Anti-mouse IgG (H + L) DyLight 600 Secondary (Cell Signaling Technologies, Danvers, MA, United States). Blots were then visualized with Odyssey Infrared Imaging System (LI-COR Biosciences, Lincoln, NE, USA). Antibodies used were pSTAT3^Y705^ (#9145), STAT3 (#8768), pAKT^Ser473^ (#9271), AKT (#9272), pFOXO^Ser253^ (#9466), FOXO3a (#12829), pFOXO1^Ser256^ (#9461), FOXO1 (#2880), OPA1 (#80471), VDAC (#4866) and Ubiquitin (#3933) from Cell Signaling Technologies. PGC-1α (#AB3242) from EMD Millipore, and COX-IV (33985) from Abcam. Optical density measurements were taken using the Image Lab Software (Bio-Rad, Hercules, CA, United States). Quantification of protein expression was measured relative to the total protein in each lane (i.e., stain-free blot) and for phosphorylation targets was quantified relative to the respective total protein. Generally, primary antibody dilution was 1:1,000, whereas the secondary antibody dilution was 1:5,000.

### 2.3 Real-time quantitative polymerase chain reaction

Total RNA from quadriceps was isolated using the miRNeasy Mini kit (Qiagen, Valencia, CA, United States) and following the protocol provided by the manufacturer. RNA was quantified by using a Synergy H1 spectrophotometer (BioTek, Winooski, VT, United States). Total RNA was reverse transcribed to cDNA by using the Verso cDNA kit (Thermo Fisher Scientific, Waltham, MA, United States). Transcript levels were measured by Real-Time PCR (7,500 Fast Real-Time PCR System: Applied Biosystems, Waltham Massachusetts) using the TaqMan gene expression assay system (Life Technologies, Carlsbad, CA, United States). Expression levels for Atrogin-1 (Mm00499523_m1), and MuRF-1 (Mm01185221_m1) were detected using Taqman Probes. Gene expression was normalized to TATA-binding protein (TBP) (Mm01277042_m1) levels using the standard 2^−ΔCT^ methods.

### 2.4 *Ex vivo* muscle contractility

Extensor digitorum longus muscles were subjected to whole-muscle contractility assessment, as we showed previously (26). The EDLs were dissected, and stainless-steel hooks were tied to both tendons using 4–0 silk sutures. The muscles were placed between a mounted force transducer (Aurora Scientific Inc., Aurora, Canada) and incubated in a stimulation bath containing Tyrode solution (121 mM NaCl, 5.0 mM KCl, 1.8 mM CaCl_2_, 0.5 mM MgCl_2_, 0.4 mM NaH_2_PO_4_, 24 mM NaHCO_3_, 0.1 mM EDTA, and 5.5 mM glucose) supplemented with continuous O_2_/CO_2_ (95/5%). Force data was collected and analyzed with the Dynamic Muscle Control/Data Acquisition and Dynamic Muscle Control Data Analysis programs (Aurora Scientific Inc., Aurora, Canada) and EDL muscle weight and L0 were used to determine specific force.

### 2.5 Statistics

Statistical analyses were performed using GraphPad Prism 9.4.1 (GraphPad Software, San Diego, CA, United States). A one-way ANOVA was employed to determine differences between groups and assess effectiveness of the MOTS-c, Bonferroni’s *post hoc* comparisons was used. In general, variance was tested throughout our study, and in most cases, there were no significant differences. When a variance was significant, a Welch’s test was used. Statistical significance was set at *p* < 0.05, and data were each presented as mean ± standard deviation (SD), unless otherwise noted.

## 3 Results

### 3.1 MOTs-c upregulates markers associated with mitochondrial biogenesis *in vitro*

To evaluate the direct effects of MOTS-c on skeletal muscle cells, fully differentiated myotubes (day 5) were treated with 50 μm MOTS-c for 24 h. MOTS-c significantly increased the mRNA expression of PGC-1α and PGC-1β (+84.6% and +80.7%, respectively; *p* < 0.05) (Supplementary Figures S1A–B). In parallel, MOTS-c enhanced the phosphorylation (at Thr172) of AMPK (+103.1%, *p* < 0.05) (Supplementary Figure S1C), indicating activation of a canonical upstream signaling pathway that promotes mitochondrial remodeling. Altogether, these findings demonstrate that MOTS-c initiates molecular signaling events consistent with stimulation of mitochondrial biogenesis and metabolism in skeletal muscle cells.

### 3.2 MOTS-c does not attenuate body weight or fat mass loss observed in C26 tumor hosts

**Figure 1:**
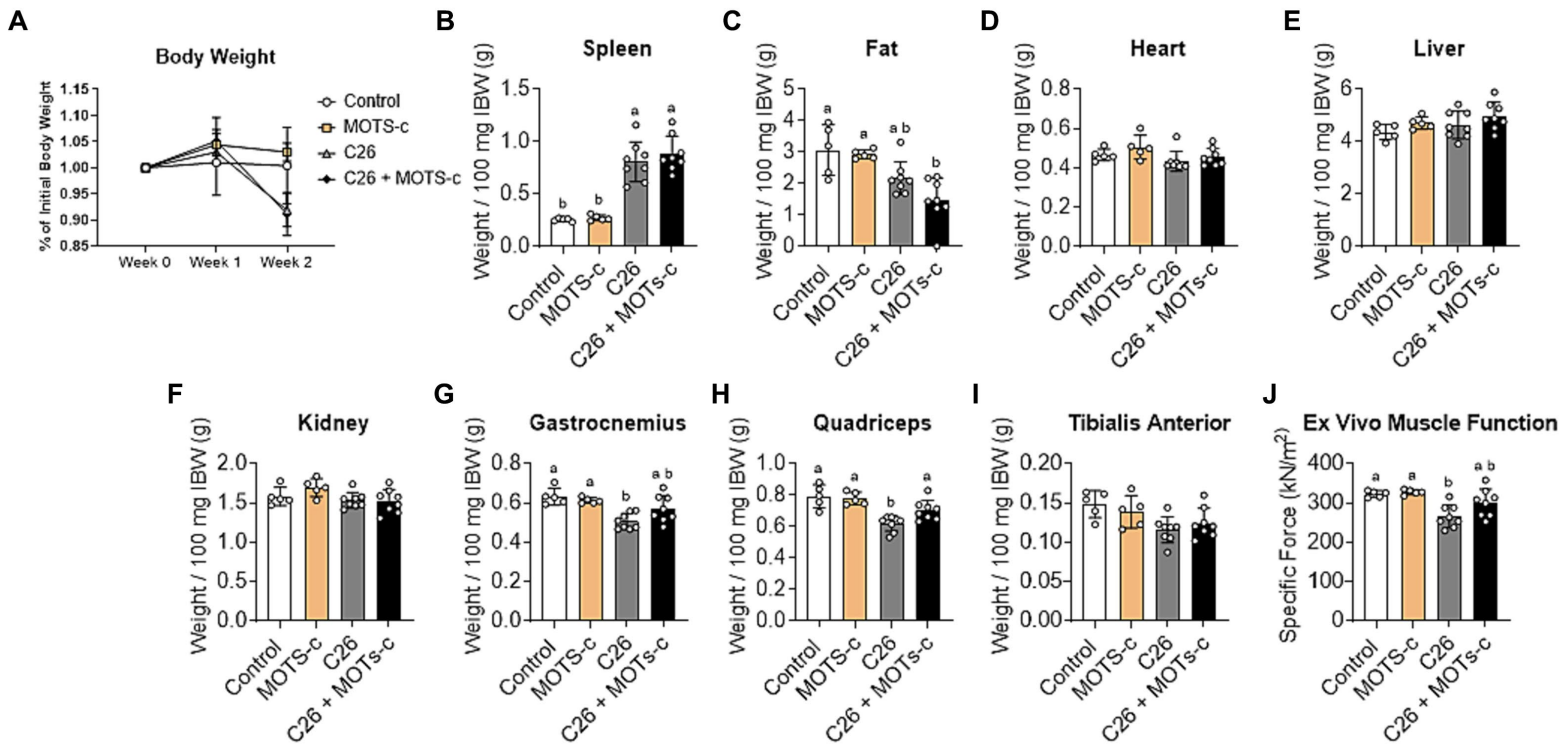
**(A)** Body weight, **(B)** spleen, **(C)** fat, **(D)** heart, **(E)** liver, and **(F)** kidney, (**G)** gastrocnemius, **(H)** Quadriceps, **(I)** tibialis anterior muscle. Weights were normalized to initial body weight (IBW) (weight•100 mg. IBW^−1^). **(J)** EDL *ex-vivo* force measured between 10–150 Hz. **(E)** Peak *in vivo* plantar flexion torque. *Ex vivo* force expressed as kN/m^2^. *In vivo* torque expressed as mN•m. Data expressed as mean ± SD. Different letters denote significant differences: *p* < 0.05.

By using the C26 model for the study of cancer cachexia, we observed that final body weight was markedly reduced in C26 tumor-bearing mice compared with controls, and this reduction was not mitigated by MOTS-c administration [one-way ANOVA: *F*(3, 22) = 11.77, *p* < 0.0001]. Both untreated C26 hosts and C26 hosts administered MOTS-c (C26 + MOTS-c) exhibited significantly reduced FBW compared with controls (−8.1% and −8.9%, respectively; *p* < 0.05), with no differences between tumor-bearing groups (Figure 1A).

Consistent with tumor burden, spleen mass was also significantly affected by treatment [*F*(3, 22) = 33.83, *p* < 0.0001]. Both C26 and C26 + MOTS-c groups showed markedly increased spleen mass relative to controls (+217% and +225%, respectively; *p* < 0.05), with no significant differences between tumor-bearing groups (Figure 1B).

Similarly, fat mass also differed across groups [*F*(3, 22) = 9.42, *p* = 0.0003]. A significant reduction in fat mass was observed in the C26 + MOTS-c hosts (*p* < 0.05, −29.2%, vs. controls), whereas fat mass in C26 hosts did not differ significantly from either controls or C26 + MOTS-c mice (Figure 1C). Despite this reduction, fat mass did not differ between the two tumor-bearing groups, suggesting that MOTS-c does not confer protection against tumor-induced adipose tissue loss.

In contrast, heart [*F*(3, 22) = 2.35, *p* = 0.10], liver [*F*(3, 22) = 2.032, *p* = 0.14], and kidney [*F*(3, 22) = 2.36, *p* = 0.09] masses were not significantly altered by C26 tumor burden or by MOTS-c administration. Importantly, MOTS-c treatment alone had no detectable effect on body weight or organ mass in non-tumor bearing mice (Figures 1D–F).

### 3.3 MOTS-c attenuates skeletal muscle mass loss and *ex vivo* skeletal muscle function deficits in C26 tumor hosts

C26 tumor burden was associated with marked skeletal muscle atrophy, which was partially attenuated by MOTS-c administration in a muscle-specific manner. Gastrocnemius mass differed significantly across groups [*F*(3, 22) = 8.67, *p* = 0.0005]. Specifically, the C26 hosts exhibited significantly reduced gastrocnemius mass compared with controls (−20%, *p* < 0.05). In contrast, gastrocnemius mass in C26 + MOTS-c hosts did not differ from either C26 or control mice, suggesting an intermediate phenotype without full preservation of muscle mass (Figure 1G).

Quadriceps muscle mass more robustly affected by tumor burden and MOTS-c treatment [*F*(3, 22) = 12.86, *p* < 0.0001]. C26 hosts exhibited substantial quadriceps atrophy relative to controls (−22%, *p* < 0.05), whereas quadriceps mass was significantly greater in C26 + MOTS-c mice compared with untreated C26 hosts (−12%, *p* < 0.05) (Figure 1H). Notably, quadriceps mass in C26 + MOTS-c mice was comparable to that of controls, indicating partial protection by MOTS-c (Figure 1H).

In contrast, tibialis anterior mass was not significantly altered by C26 tumor burden or MOTS-c treatment [*F*(3, 22) = 1.78, *p* = 0.18] (Figure 1I).

To assess whether changes in muscle mass translated to functional outcomes, *ex vivo* contractile properties were evaluated in isolated extensor digitorum longus (EDL) muscles. While treatment groups generally differed significantly [*F*(3, 22) = 7.53, *p* = 0.0012], C26 tumor-bearing mice demonstrated reduced maximal force production compared with controls (−18%, *p* < 0.05), consistent with impaired muscle function (Figure 1J). However, EDL force production in C26 + MOTS-c hosts did not differ from either C26 hosts or controls, suggesting only a modest or incomplete functional rescue that paralleled the partial preservation of muscle mass. Importantly, MOTS-c administration in non-tumor-bearing mice did not affect skeletal muscle mass or *ex vivo* muscle function, indicating that MOTS-c does not induce hypertrophy or functional enhancement under basal conditions.

### 3.4 MOTS-c attenuates increased expression of makers associated with skeletal muscle atrophy and downregulation of mitochondrial biogenesis in C26 tumor hosts

To investigate whether MOTS-c modulates molecular pathways implicated in cancer-induced muscle wasting, key signaling proteins and markers of proteolysis and mitochondrial biogenesis were examined in skeletal muscle. C26 tumor burden was associated with robust activation of inflammatory and catabolic signaling pathways that were only partially modified by MOTS-c administration.

**Figure 2:**
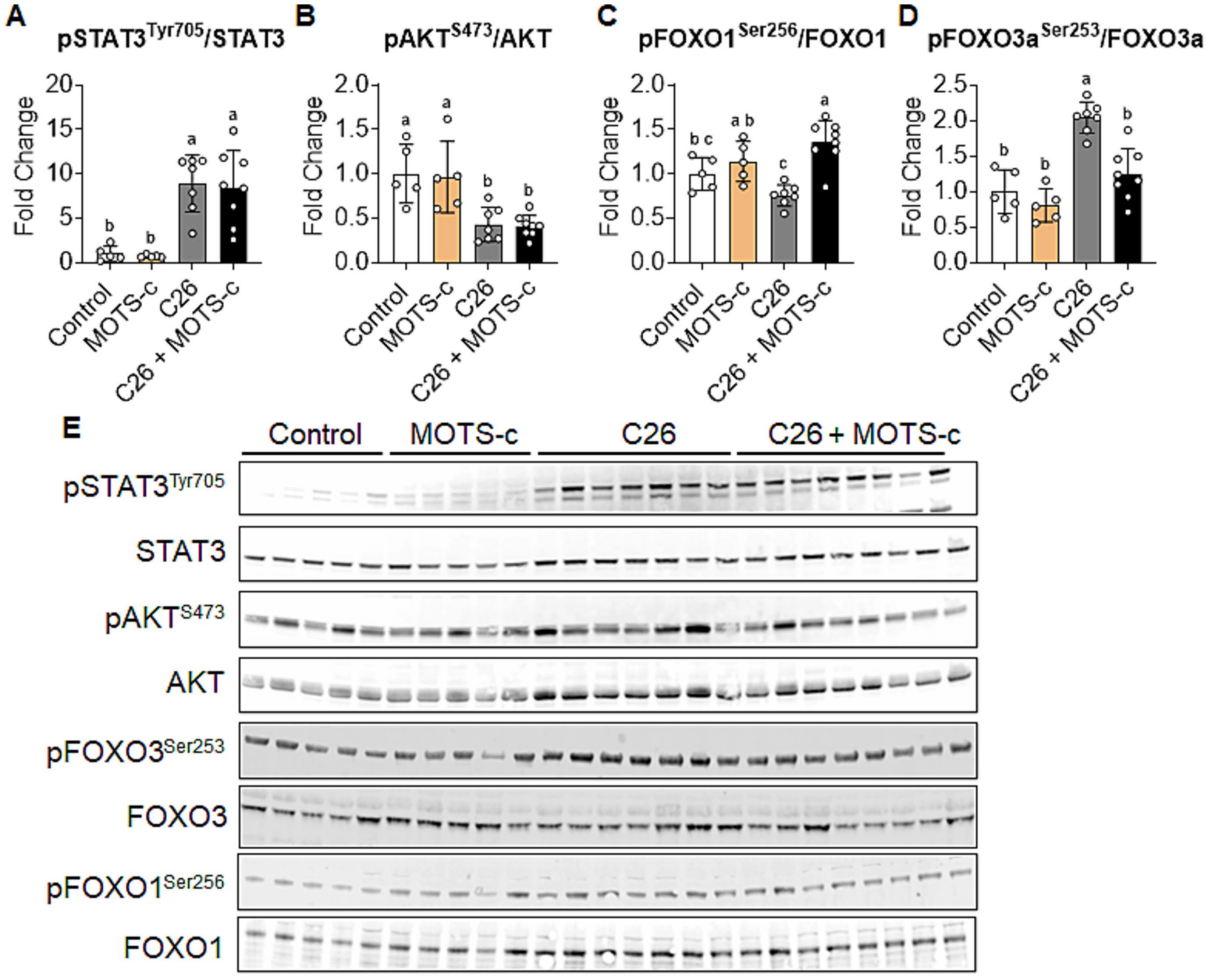
Protein expression (assessed via Western blotting) of **(A)** pSTAT3^Tyr705^, **(B)** pAKT^Ser473^, **(C)** pFoxO3a^Ser253^, **(D)** pFoxO1^Ser256^. **(E)** Representative western blottings. Protein quantification was reported as fold-change vs. control. Data expressed as mean ± SD. Different letters denote significant differences: *p* < 0.05.

Phosphorylated STAT3 (Tyr705) expression differed significantly across groups [*F*(3, 21) = 13.74, *p* < 0.0001]. Both C26 and C26 + MOTS-c mice hosts displayed markedly elevated pSTAT^TYR705^ levels compared with controls (+789% and +745%, respectively; *p* < 0.05), with no differences between the tumor-bearing group (Figure 2A). Similarly, phosphorylated AKT (Ser473) was modulated across treatments [*F*(3, 21) = 9.40, *p* = 0.0004], with both C26 and C26 + MOTS-c mice showing significant reductions in pAKT (−57% and −59% vs. controls; *p* < 0.05), whereas no differences between tumor groups were observed (Figure 2B).

In contrast, components of the FOXO signaling pathway exhibited differential regulation in response to MOTS-c. Phosphorylated FOXO1 (Ser256) was significantly affected by treatment [*F*(3, 21) = 11.66, *p* = 0.0001]. C26 + MOTS-c mice exhibited elevated pFOXO1 levels compared with controls (+36%, *p* < 0.05), whereas C26 hosts did not differ from controls. Levels of pFOXO1 were significantly higher in C26 + MOTS-c mice compared with C26 hosts (+80%, *p* < 0.05) (Figure 2C). Phosphorylated FOXO3a (Ser253) also varied significantly by group [*F*(3, 21) = 21.40, *p* < 0.0001] and was increased in C26 hosts relative to controls, consistent with activation of catabolic transcriptional programs. Notably, FOXO3a phosphorylation in C26 + MOTS-c mice was reduced relative to untreated C26 hosts (−39%, *p* < 0.05) and was comparable to control levels, indicating partial attenuation of tumor-induced FOXO3a signaling (Figure 2D).

**Figure 3:**
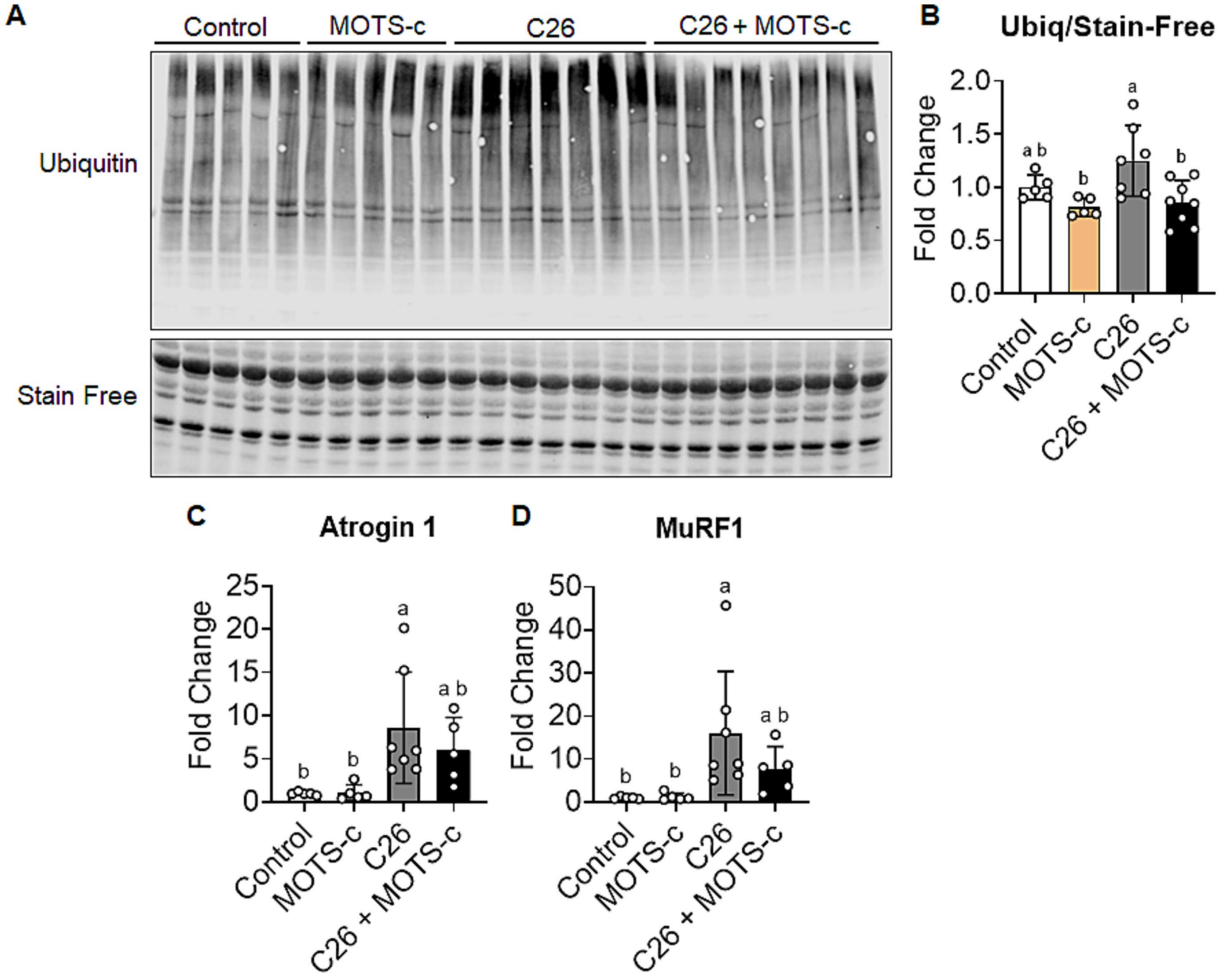
**(A)** Representative immunoblot of total ubiquitinated proteins in control, MOTS-c, C26, and C26 + MOTS-c groups. Stain-free total protein is shown below as a loading control. **(B)** Quantification of total ubiquitin normalized to stain-free total protein and expressed as fold change relative to control. Relative mRNA of the E3 ubiquitin ligases **(C)** Atrogin-1 and **(D)** MuRF1 across experimental groups, expressed as fold change relative to control. Data are presented as individual data points with bars representing mean ± SD. Groups not sharing a common letter are significantly different from one another (*p* < 0.05).

Markers of proteasome-mediated protein degradation were also altered by tumor burden and MOTS-c treatment [*F*(3, 21) = 5.16, *p* = 0.0079]. Total ubiquitin protein expression was elevated in C26 hosts (+25% vs. controls, *p* < 0.05), while the C26 + MOTS-c mice showed significantly lower ubiquitin expression compared with C26 hosts (−32%, *p* < 0.05) (Figure 3B). Consistent with these findings, expression of the muscle-specific E3 ubiquitin ligases Atrogin-1 and MuRF1 was markedly increased in C26 hosts vs. controls (Atrogin-1: +8.6-fold, *p* < 0.05; MuRF-1: 16-fold, *p* < 0.05). In contrast, expression of both atrogenes in C26 + MOTS-c mice did not differ significantly from control levels, although they were not significantly reduced compared with untreated C26 hosts, suggesting partial or variable suppression of the atrophy program (Figures 3C,D).

Consistent with previous reports showing an involvement of mitochondrial turnover in skeletal muscle in cancer cachexia (6, 25, 27), markers of mitochondrial biogenesis were also assessed. PGC-1α protein levels differed significantly across groups [*F*(3, 21) = 5.05, *p* = 0.0086] and were substantially reduced in C26 hosts (−54%, *p* < 0.05). This decline was partially rescued in C26 + MOTS-c mice, which showed significantly higher PGC-1α levels compared with C26 hosts (+143%, *p* < 0.05) and no difference in the controls (Supplementary Figure S2A). Other mitochondrial markers, including COX-IV, VDAC, and OPA1, were unchanged across groups (Supplementary Figure S2B,D).

## 4 Discussion

Mitochondrial-derived peptides have emerged as promising therapeutic candidates for metabolic and musculoskeletal disorders, yet their efficacy in severe, inflammation-driven muscle wasting remains unclear. Here, we evaluated whether MOTS-c could mitigate skeletal muscle defects in the aggressive C26 tumor model of cachexia. Although MOTS-c did not prevent systemic weight loss, it partially preserved skeletal muscle mass and *ex vivo* muscle function, supporting a protective, albeit incomplete, role in this context.

Consistent with previous reports (6, 25, 27), C26 tumor growth induced significant whole-body weight loss that was not attenuated by MOTS-c treatment, reflecting the severity and inflammatory burden of this model. Importantly, despite equivalent reductions in total body weight between C26 and C26 + MOTS-c groups, MOTS-c treatment resulted in partial preservation of quadriceps and gastrocnemius mass, indicating a dissociation between changes in whole-body mass and skeletal muscle preservation. This apparent discrepancy is best explained by the relatively small contribution of individual skeletal muscles to total body mass. Indeed, the muscles quantified in this study accounted for only ~1.3–1.5% of total body weight, such that even biologically meaningful protection of muscle tissue would be insufficient to measurably influence overall body mass. In contrast, loss of adipose tissue represents a larger determinant of body weight change in cachexia. Consistent with this interpretation, gonadal fat mass was reduced in both tumor-bearing groups, with an average raw difference of ~250 mg between treatments, an amount likely sufficient to offset the absolute preservation of skeletal muscle mass observed with MOTS-c. Thus, while MOTS-c selectively spared skeletal muscle, it did not prevent adipose tissue loss, providing a parsimonious explanation for the lack of difference in total body weight between groups.

The structural preservation of skeletal muscle with MOTS-c treatment was accompanied by functional benefits, as *ex vivo* EDL force production was impaired in C26 hosts but partially rescued by MOTS-c. These findings align with prior studies demonstrating that MOTS-c mitigates muscle atrophy in hindlimb immobilization models (17) and improves exercise capacity across the lifespan in mice (18). In humans, circulating MOTS-c positively correlates with muscle strength (28), and the K14Q MOTS-c variant (29), characterized by reduced phosphorylation-activating binding capacity, increases the risk of type 2 diabetes mellitus (30), alters skeletal muscle composition and function (31), and elevates sarcopenia risk (24). Together, these data support a conserved role for MOTS-c in maintaining muscle mass and function and position our findings within a broader translational framework. While MOTS-c did not prevent cachexia, its ability to preserve muscle mass and force output in this aggressive tumor model underscores its therapeutic potential.

Mechanistically, MOTS-c is known to regulate metabolic and anti-atrophic signaling through AKT, FOXO1/3a, GLUT4, and heat shock proteins (13, 16–18, 23), many of which are typically dysregulated in cancer cachexia. Indeed, suppression of AKT signaling is a hallmark of cachectic muscle (1, 32, 33), and restoring AKT activity can reverse cachexia phenotypes (34). Although MOTS-c enhances AKT phosphorylation in multiple atrophy and metabolic disease models (13, 16, 17), here we observed a downregulation of pAKT^Ser473^ in both C26 groups, with no rescue by MOTS-c. This divergence from prior studies likely reflects the extreme inflammatory and catabolic burden imposed by the C26 model, which may override canonical anabolic signaling mechanisms.

STAT3 is a transcription factor extensively implicated in the regulation of cancer cell proliferation, and pharmacologic inhibition of STAT3 signaling has demonstrated broad anti-cancer efficacy (35, 36). Importantly, increased STAT3 phosphorylation in skeletal muscle is a well-established feature of cancer cachexia and is largely driven by interleukin-6 (IL-6), a cytokine secreted by the tumor microenvironment (37, 38). Importantly, pharmacological or genetic inhibition of IL-6, its receptor, or STAT3 itself has been shown to significantly attenuate the severity of cachexia in tumor-bearing hosts (27, 39, 40). In the present study, STAT3 phosphorylation was markedly elevated in both C26 tumor-bearing groups, and this increase was not attenuated by MOTS-c treatment. Because no prior studies have directly evaluated whether MOTS-c influences STAT3 activation in wasting conditions, our data indicates that MOTS-c’s protective effects in the C26 hosts occur independently of STAT3 suppression. Together, these findings indicate that in severe cachexia, MOTS-c does not restore classical anabolic signaling pathways but instead confers muscle protection through alternative or compensatory mechanisms.

FOXO transcription factors are central regulators of muscle proteostasis, with reduced phosphorylation promoting nuclear localization and induction of atrogene expression (17, 41–45). MOTS-c has been shown to increase the phosphorylation of FOXO via the CK2/PTEN/AKT/FOXO signaling axis (16, 17, 24). In the present study, however, we observed divergent regulation of FOXO isoforms in C26 tumor-bearing hosts. Specifically, phosphorylation of FOXO1 at Ser256 (pFOXO1^Ser256^) was reduced in C26 hosts and attenuated by MOTS-c, consistent with suppression of FOXO1-driven atrophic signaling. In contrast, phosphorylation of FOXO3a at Ser253 (pFOX3a^Ser253^) was increased in C26 hosts and this upregulation was attenuated by MOTS-c in C26 hosts. Previous studies conducted using models of immobilization showed decreases in both pFOXO1 and pFOXO3a that were attenuated by MOTS-c (17). Thus, the present patterns may reflect isoform-specific regulation in cancer cachexia. Because AKT directly controls pFOXO1^Ser256^-mediated nuclear/cytosolic shuttling (46), its reduction in C26 hosts aligns with expected FOXO1-driven catabolism. In contrast, increased pFOXO3a^Ser253^ in C26 hosts may represent a compensatory response limiting excessive FOXO3-mediated atrophy (47, 48). Importantly, FOXO3a activity is not restricted to nuclear transcriptional control. AMPK-dependent mitochondrial translocation of FOXO3a, regulated by distinct phosphorylation sites, represents a parallel regulatory network (49). Given that MOTS-c activates AMPK (13), the reduction in pFOXO3a^Ser253^ following MOTS-c treatment may reflect a redistribution of FOXO3a toward mitochondrial functions rather than enhanced nuclear atrogene signaling. Thus, decreased pFOXO3a^Ser253^ in this context should not be interpreted as permissive of FOXO3a-driven muscle catabolism. Collectively, these findings indicate that MOTS-c modulates FOXO signaling in an isoform- and context-dependent manner, rather than uniformly restoring canonical anabolic regulatory pathways.

Activation of the ubiquitin-proteasome system (UPS) is a defining feature of cachexia-induced muscle wasting, characterized by elevated muscle-specific E3 ubiquitin ligases (e.g., MuRF1, Atrogin 1) and increased protein ubiquitination (50–54). Consistent with prior C26 studies (25, 32), we observed increased MuRF1, Atrogin1 and ubiquitin protein levels in tumor hosts. Despite the variability in E3 ligase expression was evident and likely limited statistical significance between groups, the reduction in mean expression with MOTS-c was noteworthy. Ubiquitin protein levels increased in C26 hosts but were unchanged in C26 + MOTS-c hosts when compared to controls. These findings suggest that MOTS-c partially attenuates UPS activation, aligning with the observed preservation of muscle mass and function.

Collectively, this study adds to the growing evidence supporting exogenous MOTS-c as a therapeutic candidate across different disease conditions (13–22, 24, 31, 55–58). Nonetheless, several limitations should be acknowledged. We did not compare MOTS-c with other mitochondrial-targeted therapeutics, such as MitoQ, nor did we assess food intake, which may influence cachectic outcomes. Additionally, MOTS-c administration began at tumor inoculation; because clinical cachexia is typically treated after disease diagnosis and symptom onset, future studies should evaluate the therapeutic efficacy of MOTS-c when administered after cachexia has already developed. Finally, while we acknowledge the importance of investigating the correlation between signaling modulation and tumor-induced atrophy, we did not perform *in vitro* experiments to assess the effects of MOTS-c on myotube atrophy in response to C26 tumor-conditioned media or co-culture models with cancer cells. Beyond these limitations, further investigation is warranted to elucidate the interactions between MOTS-c and mitochondrial quality control pathways, including mitophagy and biogenesis, and to define the specific signaling networks through which MOTS-c confers muscle protection in severe inflammatory environments.

In conclusion, although MOTS-c did not fully prevent the cachectic phenotype induced by C26 tumors, it provided meaningful protection against tumor-induced skeletal muscle deterioration. MOTS-c preserved muscle mass and *ex vivo* contractile function despite ongoing cachexia-related signaling disruption, including reduced AKT activation, increased STAT3 phosphorylation, and elevated UPS activity. Notably, its selective modulation of FOXO1 and FOXO3a phosphorylation suggests engagement of alternative or compensatory stress-response mechanisms rather than complete restoration of classical anabolic pathways. While modest, these effects highlight MOTS-c as a promising adjunctive strategy for attenuating cancer-associated muscle wasting and support further investigation into its therapeutic potential in cachexia.

## Data Availability

The original contributions presented in the study are included in the article/Supplementary material, further inquiries can be directed to the corresponding authors.
