# Supplementary material for: MOTS-c partially protects against skeletal muscle deterioration in C26 cachexia
Source: Front Med (Lausanne). 2026 May 25;13:1838178. doi: 10.3389/fmed.2026.1838178 (PMC13243040; doi:10.3389/fmed.2026.1838178)

Figure S1. Relative mRNA expression of **(A)** PGC-1α and **(B)** PGC-1β in Control and MOTS-c groups, expressed as fold change relative to Control. **(C)** Quantification of $pAMPK^{Thr172}$ normalized to total AMPK and expressed as fold change relative to Control. **(D)** Representative immunoblots showing phosphorylated AMPK at Thr172 and total AMPK in Control and MOTS-c groups. Data are presented as individual data points with bars representing mean ± SD. Groups not sharing a common letter are significantly different from one another ($p < 0.05$).

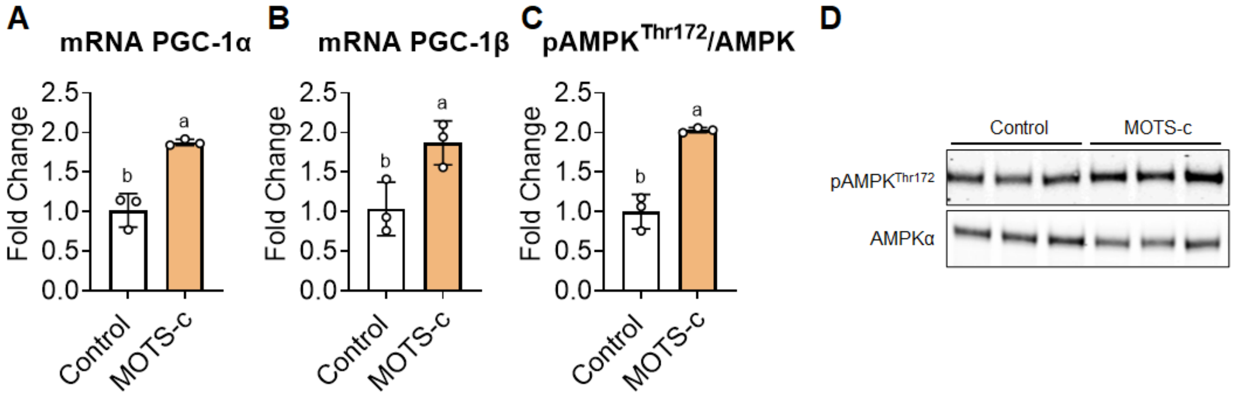

Figure S2. Quantification of protein abundance normalized to stain-free total protein for **(A)** PGC-1α, **(B)** COX IV, **(C)** VDAC, and **(D)** OPA1 in Control, MOTS-c, C26, and C26 + MOTS-c groups. Data are expressed as fold change relative to Control. **(E)** Representative immunoblots showing PGC-1α, COX IV, VDAC, and OPA1 protein expression, with stain-free total protein shown below as a loading control. Data are presented as individual data points with bars representing mean ± SD. Groups not sharing a common letter are significantly different from one another ($p < 0.05$).

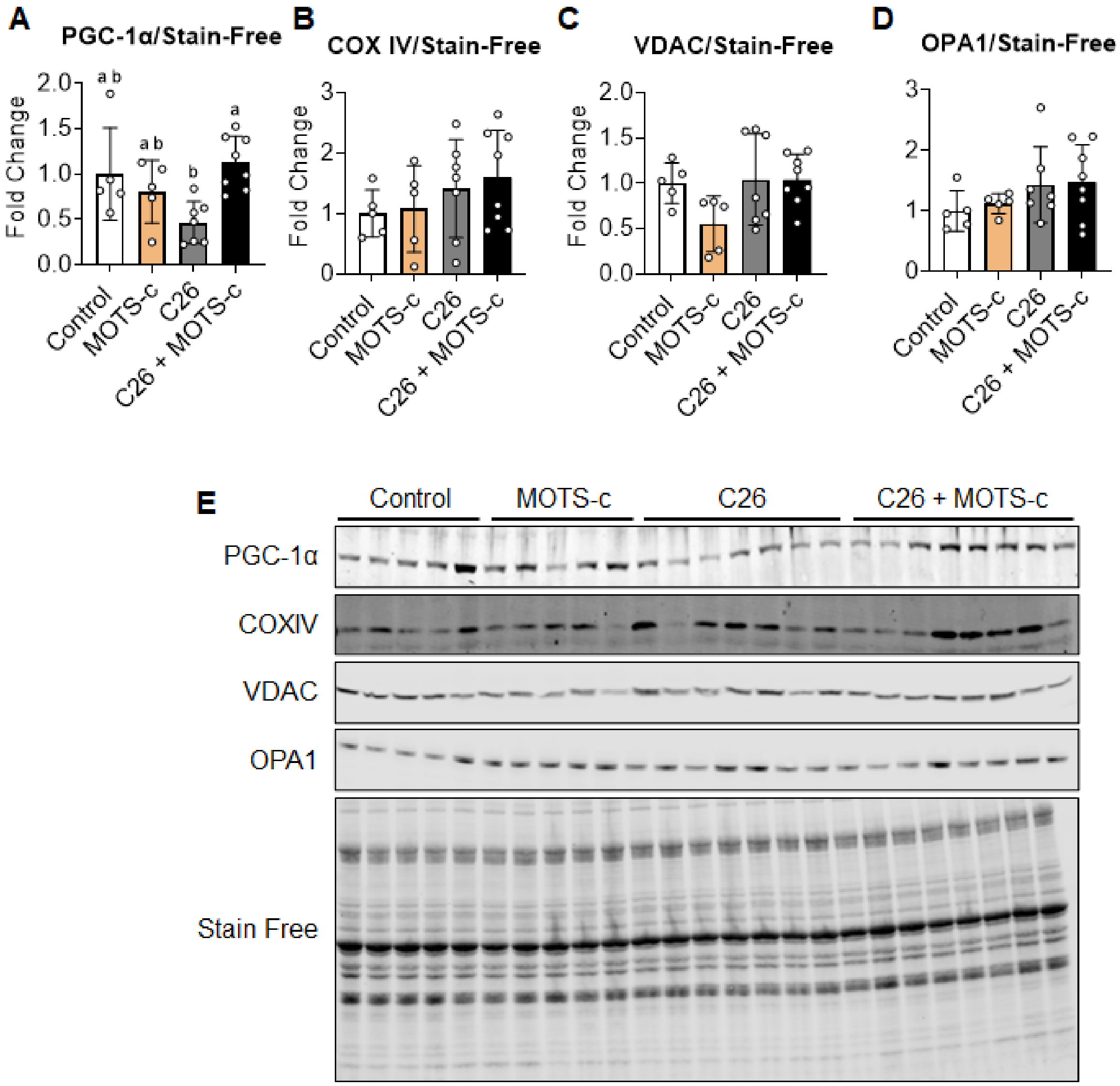

Supplement: Supplementary file 1 [file Data_Sheet_1.pdf]
